# Evaluating patient reported outcomes and experiences in a novel proton beam clinic – challenges, activities, and outcomes of the ProtonCare project

**DOI:** 10.1186/s12885-023-10586-y

**Published:** 2023-02-09

**Authors:** K Sjövall, U Langegård, P Fransson, E Nevo-Ohlsson, I Kristensen, K Ahlberg, B Johansson

**Affiliations:** 1 Faculty of Health Sciences, Kristianstad University, SE-291 88 Kristianstad, Sweden; 2 Institute of Health and Care Sciences, Göteborg University, Box 457, SE- 405 30 Göteborg, Sweden; 3 Department of Nursing, Umeå University, SE-90 187 Umeå, Sweden; 4 School of Health Sciences, Örebro University, SE-701 82 Örebro, Sweden; 5 Systemic Radiation Therapy, Lund University, SE-221 00 Lund, Sweden; 6 Blod- Och Tumörsjukdomar Administration, Uppsala University, SE- 51 85 Uppsala, Sweden

**Keywords:** Research program planning, Proton beam therapy, Patient reported outcomes, Cancer nursing, Radiotherapy

## Abstract

**Background:**

The ProtonCare Study Group (PCSG) was formed with the purpose to develop and implement a framework for evaluation of proton beam therapy (PBT) and the related care at a novel clinic (Skandionkliniken), based on patient reported data.

**Method:**

A logic model framework was used to describe the process of development and implementation of a structured plan for evaluation of PBT for all diagnoses based on patient reported data. After the mission for the project was determined, meetings with networks and stakeholders were facilitated by PCSG to identify assumptions, resources, challenges, activities, outputs, outcomes, and outcome indicators.

**Result:**

This paper presents the challenges and accomplishments PCSG made so far. We describe required resources, activities, and accomplished results. The long-term outcomes that were outlined as a result of the process are two; 1) Improved knowledge about health outcomes of patients that are considered for PBT and 2) The findings will serve as a base for clinical decisions when patients are referred for PBT.

**Conclusion:**

Using the logical model framework proved useful in planning and managing the ProtonCare project. As a result, the work of PCSG has so far resulted in long-lasting outcomes that creates a base for future evaluation of patients’ perspective in radiotherapy treatment in general and in PBT especially. Our experiences can be useful for other research groups facing similar challenges. Continuing research on patients´ perspective is a central part in ongoing and future research. Collaboration, cooperation, and coordination between research groups/networks from different disciplines are a significant part of the work aiming to determine the more precise role of PBT in future treatment options.

## Background

Radiotherapy is one of the primary modalities in cancer treatment. It is estimated that about half of cancer patients globally would benefit from radiotherapy for cure or palliation [1, 2]. The search for methods to optimize radiation dosage to kill cancer cells while limiting its effects to normal tissues, both by improvements of conventional radiotherapy (CRT) with photons and by introducing new treatment modalities, has been a major focus for decades [3].

Proton beam therapy (PBT) is increasingly utilized in cancer treatment with the hope of mitigating radiation-induced side-effects. Like intensity-modulated and image-guided radiotherapy, PBT has been shown to be a technological advance capable of improving the delivery of radiotherapy. Although the dosimetric advantages compared to CRT has been proven in numerous studies, there is a need for evaluation of patient reported outcomes. This paper describes the challenges in the development and implementation of a framework for evaluation of PBT and the related care at a novel clinic (Skandionkliniken), based on the patient reported data.

### Proton beam therapy

PBT is an advanced radiotherapy modality in which proton particles penetrate deep into the tissue and target and stop at a certain depth depending on their energy. The suggested advantages of PBT compared to CRT are based on dosimetric comparisons. PBT delivers a lower delivered dose outside of the target tissue in comparison with CRT leading to a lower risk of normal tissue toxicity [4]. PBT is increasingly utilized in cancer treatment with the hope of mitigating radiation-induced side-effects [5–7]. However, as advanced PBT is of limited capacity, it is important to evaluate and compare PBT to CRT to validate the actual gain on an individual basis. The limited data available shows that PBT is related to favorable patient reported outcomes (PRO) for brain, head-neck, lung and pediatric cancers [8]. However, studies investigating health related quality of life (HRQoL) and symptom experience in patient receiving PBT compared to modern CRT using patient reported outcomes and patient reported experiences are still lacking [9, 10].

### Patient reported outcomes and patient reported experiences

It is well recognized that patient-reported outcome measure (PROM) and patient-reported experience measures (PREM) are central in the evaluation of new treatment modalities and quality of care. PROMs are increasingly used in cancer research as patients’ treatment-related symptoms and quality of life is recognized as a central part of cancer survivorship [11, 12]. However, the evidence for the impact the treatment and the disease on of quality of life in is insufficient for some groups of patients, especially for patients with brain tumor [13, 14]. Moreover, the overall prognostic value of PROMs for survival may vary related to different diagnoses and stage of the disease [13, 14]. As for PREM, there is a positive association between patient experience and health outcomes such as adherence to recommended treatment, preventive care and resource use [15].

Beyond survival, maintenance or improvement of functioning and QoL are important outcomes of treatment. Symptoms related to radiotherapy represent a departure from the normal function or feeling, are observed by the patient, and can seldom be measured directly. Especially subjective toxicities are at high risk to be underreported by clinicians, even when prospectively collected within treatment study protocols [16]. Although evaluation of patients functioning status may be stable during and after CRT, patients’ individual and subjective evaluation of HRQoL may differ considerable during the same period [17]. Several studies have demonstrated that PROMs are the most sensitive method for capturing treatment related toxicity as clinicians’ assessment often report fewer symptoms with lower severity than patients. On the other hand, clinicians’ assessment of toxicity is needed to infer causation and to exclude issues unrelated to the treatment [16, 18]. Given that PBT has the potential to reduce toxicities compared to CRT, but still is unproven in many aspects, the combined use of patient-reported and clinician assessed outcomes is necessary in the building of evidence.

### The Skandion clinic

The construction of the Skandion Clinic was initiated in 2011 and the first patients were treated in August 2015. The Skandion Clinic is the only public proton therapy facility in Sweden, based on shared governance between all health care regions in Sweden. The clinic has an organizational model of distributed competence, building on a collaboration where all clinical experts work close to their patients in regional centers [19]. All preparations are conducted at the home clinic for all patients, including the dose planning. After completion of PBT, the patient is re-referred to the home clinic. At least 80% of the patients receiving therapy at the Skandion clinic are expected to be included in a clinical prospective PBT protocol. From the planning and start of the clinic it has been an expressed purpose to show how great the advantages of PBT are compared to CRT [20]. The project group working in the preparation phase was formed by physics and medical expertise in PBT and CRT, who represented the seven university hospitals sharing the governance of the Skandion clinic [19]. They formed the base for the diagnosis-related study protocol groups.

### Startup of the The ProtonCare Study Group (PCSG)

PCSG was established as a result of an initiative of researchers specialized in oncology nursing. The initiative also resulted in a commission from the Skandion management to form a research group conducting caring research in conjunction with PBT. The overall purpose of the ProtonCare project is to evaluate PBT and PBT related care from the patients’ perspective by assessing patient reported outcomes and experiences in patients undergoing PBT at the Skandion Clinic or CRT at conventional RT departments. The ProtonCare project started in 2013 with the formation and start-up of PCSG. In this paper we describe the process for establishing a national research group, appropriate networks and a framework for evaluating patients experiences of proton beam therapy at the novel clinic.

## Method

Inspired by the logic model framework developed by Hayes, Parchman and Howard [21], we describe the process of establishing a research network for patient experiences related to PBT. Well-designed activities directly linked or relevant to the outcomes of the project are crucial and an essential element in a logic model development. A logical model is a framework for describing the resources, activities, and desired results which can be helpful in project management, resource allocation and strategic planning. The process of developing the logic model facilitates critical thinking through the process of planning and communicating objective and outcomes [21, 22] and raise awareness and understanding of the challenges ahead [23].

The first step in the model was to define and agree on the mission for the ProtonCare project and describe the underlying assumptions.

In the second step resources and challenges were identified and defined. Thereafter activities to fulfill the mission and meet the needs of the assumption were described and planned.

In step three, all the elements of the logic model are eventually defined, as the specific outputs and outcomes necessary are identified and gradually to some extent fulfilled.

## Result

The process is described in the three steps below and is summarized in Table 1.

**Table 1:** ProtonCare Study Group (PCSG) logic model

Assumptions	Resources and challenges	Activities	Outputs	Outcomes
Lack of clinical data demonstrating superior outcomes in the use of PBT over CRT Lack of evidence regarding patient reported toxicity and patients experiences related to PBT, hence a need for research based on PROs Evaluation of patients´ satisfaction with care is central when starting up a novel clinic	**Resources** - The establishment of the PCSG and as a result a commission from the Skandion management to conduct caring research in conjunction with PBT **Constraints** - Lack of infrastructure for research at the Skandion clinic **Challenges** - Staffing - Funding - Potential study participants geographically spread - Limited number of patients referred for PBT - Diagnosis related study protocols not completed from startup but evolving continuously - Evolving data collection implementation for patients treated with PBT, in concordance with each diagnose based protocol group - Data collection implementation for patients treated with CRT	Convene PCSG meets Attend diagnosis study protocol meets Applying for funding for support of the PCSG Applying for funding to support PCSG research projects Hire research nurses and doctoral students Setting up a structure for data collection Creation of a network of coordinators for patient inclusion to PCSG research project Convene and host national meeting for coordinators from the Skandion clinic and participating regional radiotherapy departments Nordic collaboration on patient perspective in PBT	Extent of external funding to support PCSG meets Extent of external funding for research projects Database for collected data Dissemination of research findings by publications, communication of findings and presentation at national and international conferences Ongoing and continuous dialogue with the staff at the Skandion clinic to develop the care	**Short term** - Development of appropriate, relevant, and sustainable research network covering participating regions and radiotherapy departments - Granted funding **Intermediate** - Scientific publications - Granted funding - Use of the research findings in the quality improvement process of the care at the Skandion clinic - An increase in the number of research projects of PBT based on PROs **Long-term** - Knowledge about health outcomes of patients considered for PBT will improve - Findings will serve as base for clinical decisions when patients are referred to PBT versus CRT

### Step 1 – Agreeing on the mission and describing assumptions

Initiated by a group of researchers in caring science related to radiotherapy, the PCSG was constituted and established. The frame and the definition of the relevant research questions was based on what was previously published on patient reported outcomes and care in in conjunction with PBT. From the first meeting with the members of PCSG in 2013 the underlying assumption for the work in the group has been the need for evidence of PBT based on patient reported outcomes and patient experiences. Although the dosimetric benefits of PBT since long are well established, there is a lack of clinical data demonstrating a clear improvement in patient outcomes when comparing PBT with CRT. There is also a lack of evidence regarding patient reported experiences related to PBT. Thus, the underlying assumption for PCSG has been that patient reported outcomes and evaluation of patients´ satisfaction with care is central when implementing the new treatment modality and starting up the novel clinic. The mission was presented and communicated at regular national meetings with the diagnosis related study protocol groups for proton beam therapy.

### Step 2 – Identifying and describing resources, challenges, and activities

Meetings on a regular basis with PCSG was facilitated to identify and describe resources, challenges, and activities. A side benefit from that work was the continuous development in the new team relationship and of the focus of PCSG.

#### Resources

The initial resources for the ProtonCare project were the formation and the composition of PCSG. The PCSG is a multi-professional group with a core of researchers within caring sciences that cover different knowledge areas and professions from different universities across the country and the seven university hospitals, which share the governance of the Skandion clinic. The group members have a long experience from research within caring sciences in cancer care including patient reported outcomes in conjunction with radiotherapy as well as a broad knowledge in both qualitative and quantitative different research methods. Since the start of PCSG, resources have been developed by creating networks and building collaboration. Working closely together with the health care staff at Skandion has been of importance through the whole process. Networks have been built up to facilitate data collection (described under Activities) and for the collaboration with other study protocol groups at the Skandion clinic, e.g. the Proton Radiotherapy for Primary Central Nervous System Tumours in Adults—a Prospective Swedish Multicentre Study [24]. Several of our studies are conducted in collaboration with physics and medical expertise in PBT and CRT, and health economic expertise.

#### Challenges

### Funding and staffing

Although research and evaluation of PBT was a stated goal for the Skandion clinic, research funds have not been allocated for the purpose nor for staff recruiting study participants. Thus, to be able to perform the planned studies we needed to apply for funding. Similarly, time for the members of the PCSG had to be created. The search for funding is ongoing, as collection of data is continuing, and new research questions are evolving.

### Collaboration with evolving study protocol groups

The gold standard for the development of medical evidence regarding effects of treatments are RCTs´. However, for evaluating PBT it might not always be the scientific appropriate or ethical design to choose [25]. Alternative approaches e.g. prospective observational cohort studies for establishing evidence about the different treatment options are needed for follow-up of patients who are not included in RCTs. A primary strategy for the ProtonCare project was to evolve data collection implementation for patients treated with PBT, in concordance with each diagnose based protocol group. However, diagnosis related study protocols were not completed from the start of the clinic but are evolving continuously. New protocols are and will be developed and implemented over several years ahead. Ensuring the prospective data collection and adapting to several evolving study protocols is a continuous work.

### Recruitment of study participants

Recruitment of participants is challenging in that they are geographically spread over all Swedish health care regions. The study settings for the ProtonCare studies are several radiotherapy clinics/departments: the Skandion Clinic in Uppsala together with the radiotherapy departments at the university hospitals in Lund, Göteborg, Linköping, Örebro, Stockholm, Uppsala, and Umeå. As routines for the PBT referring process differ between the departments, ensuring the recruitment process has been a challenge.

### Data collection

A challenge with the data collection was to find a strategy able to manage the nationwide data collection in a rational and cost-effective way that at the same time as it would be adapted to the patients' different preferences. Patient-reported data is primarily collected by paper questionnaires. A web-based questionnaire was tested and evaluated as a choice for participants to respond in when preferred. Qualitative interviews face to face and by telephone are used to collect qualitative data on patients’ experiences.

For the majority of clinical situations, the prospective collection of clinical data with patient reported QoL outcomes are needed. As the diagnosis related study protocols for patients treated with PBT at the Skandion were not completed at the start-up but has evolved continuously, our data collection has evolved meanwhile in concordance with each diagnosis group. To ensure the quality of data and at the same time ensure not to burden study participants more than necessary has been a challenge in the project. To measure PROs, both disease-specific and general PROMs are used, such as QLQ-C30 [26] and EQ5D (ref) for HRQoL, MFI-20 [27] for fatigue, HADS [28] for anxiety and depression, ISI [29] for sleeping difficulties and RSAS [30] for the daily assessment of symptoms. To measure the PREMs regarding quality of care and experiences of treatment qualitative interviews together with the Quality from the Patients Perspective questionnaire [31] are used.

#### Activities

Creating and collaborating with multi-professional networks with health care professionals and researchers has been an important focus in creating activities. To meet the challenges of recruiting participants geographically spread, a network was created for coordinators at each radiotherapy department in the six health care regions together with health care professionals at the Skandion clinic. The network was based on contacts nominated by each department for the administration of patient referral. Monthly, digital meetings with the network have focused on quality assurance of the recruitment process and feedback on the progress of the studies. In addition, the network met at the Skandion clinic once a year.

As the members of PCSG are geographically spread across the country, monthly digital meetings as well as physical meetings every sixth months with the group has been the foundation for the strategic planning. A major part of the activities in PCSG have been to apply for external grants to support the work in the group and with the network, and also to apply for the funding of doctoral students and research nurses.

As facilities for PBT are nationally and globally relatively few, international collaboration is necessary to build the clinical evidence for the effect. A Nordic work-shop on the subject of “*Patients perspective in proton beam therapy”* was held in Uppsala for the first time in autumn 2019, with members from Finland, Norway, Denmark and Sweden [32]. Future annual meetings are planned to collaborate within the field.

### Step 3 – Identifying outputs, outcomes, and outcome indicators

#### Outputs

Funding was received 2015 from the Sahlgrenska Academy, Göteborgs University for a PhD-student. Another grant was received from the Swedish Cancer Society, 2017–2019 and 2020–2022. Smaller grants from local funds have contributed to enabling meetings and time for members of PCSG.

As to date, collaboration with five diagnosis groups is established. When study protocol evolves, measurements/instruments, patient information, recruitment, data collection, analysis, and publication of results is discussed in collaboration between the diagnosis group and PCSG.

During the first five years (2015–2020) at Skandion, 949 adult Swedish patients were referred and treated, of which 352 was included in studies by PCSG [33]. About one third have completed the three-year follow-up. Analysis of collected data is continuously ongoing.

#### Outcomes

### Short-term outcomes

The short—term outcomes are most closely associated with the outputs. So far, the most significant short-term outcome has been the granted funding described above. The funding has allowed for the development of appropriate, relevant, and sustainable research network covering participating regions and radiotherapy departments.

### Intermediate outcomes

Intermediate outcomes result from the application of the short-term outcomes, e.g. scientific publications. A PhD thesis was defended in February 2020 [34]. To date, PCSG has published 8 studies [35–40], presented in Table 2. As a result, there is an increase in the number of research projects of PBT based on PROMs. Another intermediate outcome is the use of the research findings in the quality improvement process of the care at the Skandion clinic, which has been a result of the continuous dialogue with the staff at the clinic.

**Table 2:** ProtonCare Study Group (PCSG) scientific publications to date

Topic	Study design	Respondent	Scientific publication
Quality of care and HRQoL during PBT	Prospective, longitudinal, descriptive, quantitative study	Patients treated with PBT for brain tumor (*n* = 186)	Langegård et al. 2018 [35]
Experiences of symptom management during PBT	Prospective, longitudinal, qualitative study	Patients treated with PBT for brain tumor (*n* = 22)	Langegård et al. 2019 [36]
Symptom cluster during PBT	Prospective, longitudinal, explorative, quantitative study	Patients treated with PBT for brain tumor (*n* = 187)	Langegård et al. 2019 [37]
Development and initial psychometric evaluation of a radiotherapy-related symptom assessment tool, based on data from patients with brain tumours undergoing proton beam therapy	Prospective, longitudinal and quantitative design	Patients treated with PBT for brain tumor (*n* = 234)	Langegård et al. 2020 [30]
Experiences of living away from home when treated with PBT	Prospective, longitudinal, qualitative study	Patients treated with PBT for brain tumor (*n* = 22)	Möllerberg et al. 2020 [39]
Patients’ perspective in the context of proton beam therapy: summary of a Nordic workshop	Summary of workshop		Ohlsson-Nevo et al. 2020 [32]
Symptom experiences and HRQoL	Prospective, longitudinal, explorative, quantitative study	Patients treated with PBT for brain tumor (*n* = 266)	Langegård et al. 2021 [38]
Evaluations of skin reactions during PBT – comparing patient-reported vs clinician reported outcomes	Retrospective, quantitative study	Patients treated with PBT for brain tumor (*n* = 253)	Möllerberg et al. 2021 [40]

### Long-term outcomes

The long-term outcomes of this project are twofold: (1) patient reported outcomes and patients experiences related to PBT will improve and (2) our study findings can serve as base for clinical decisions when patients are referred to PBT versus CRT. Development of appropriate, relevant, and sustainable research network covering participating regions and radiotherapy departments have been an ongoing process. The long-term follow-up of study-participants will last for five years post treatment. Collected data will serve as an important base for the achievement of the two main long-term outcomes. As for the two main long-term outcomes, the work is continuing.

## Discussion and concluding our experiences so far

Incorporating the patient´ perspective is critical when evaluating the treatment, clinical care and quality performance management in new PBT departments. Radiotherapy continues to evolve at a rapid rate in technology and techniques, with both driving up costs in an era in which health care budgets are of increasing concern. The more precise role of PBT in future treatment options is yet to be determined, as well as the question of equal availability for all. Thus, continuing research on patients´ perspective in PBT is a central part. Reflecting on accomplishments and challenges we´ve met has been an important reconciliation to proceed further.

For the feasibility of the ProtonCare project, one of the most significant outputs have been the granted funding so far. This has enabled the recruiting of a doctoral student and the building of the necessary structure and logistics to achieve the goals of the project, gradually rendering in publication of the research. Creating and meeting with the network for coordinators at each participating radiotherapy center was an essential part of this work, alongside with the close collaboration with the nursing staff at the Skandion clinic.

Securing the inclusion of study participants, the quality of data collection and minimizing the burden of surveys on participants have been a challenge. The need for both time and facilities for collaboration between different protocol groups with different purposes and interests has become clear during the process. Considering also the geographically spread sites referring for PBT, it has underlined the importance of a research infrastructure when implementing a new treatment method as PBT (new method in terms of availability).

A major constraint related to the resources has been the lack of infrastructure for research at the Skandion clinic from the start. The need for coordination of different study protocols and quality assurance of the recruitment process are examples of areas that would benefit from such infrastructure. On a national level, the lack of an accessible IT-platform within cancer care and research for managing data from our studies and from existing quality register have meant further challenges. One approach to solve the handling of longitudinal data was the web-based questionnaire, which was tested and evaluated as a choice for participants to respond in. However, due to rising and substantially high costs, the web-based alternative was not an alternative in the longer run.

The number of patients referred for PBT have been less than estimated from startup. For several reasons, the Skandion Clinic has not yet reached its full capacity. One is that the parallel development of CRT has reduced the need for PBT, another that the role of PBT within cancer treatment is not yet fully evident nor established (Striem 2021). Accordingly, the base for recruitment of participants to the ProtonCare projects has been smaller than first estimated, but nevertheless crucial within the continuous work of establishing the evidence of PBT.

## Conclusion

Several challenges were met during the progress of the ProtonCare project. For future PBT centra, infrastructure for research is of high importance to facilitate the research needed to create the evidence of PBT. However, several accomplishments were also made, resulting in long-lasting outcomes that creates a base for future evaluation of patients’ perspective in radiotherapy treatment in general and in proton beam therapy especially. Our experiences might be useful for other research groups facing similar challenges. Continuing research on patients´ perspective is a central part in ongoing and future research. Collaboration, cooperation, and coordination between research groups/networks from different disciplines are a significant part of the work aiming to determine the more precise role of PBT in future treatment options.

## Data Availability

The datasets generated during the current study are not publicly available as the generation of data is still ongoing. Data will be available from the corresponding author on reasonable request.

## Abbreviations

PBT: Proton beam therapy
CRT: Conventional radiotherapy
PRO: Patient reported outcomes
HRQoL: Health related quality of life
PROM: Patient reported outcome measures
PREM: Patient reported experience measures
PCSG: The ProtonCare Study Group
